# Diagnostic Value of Contrast-Enhanced Dual-Energy Computed Tomography in the Pancreatic Parenchymal and Delayed Phases for Pancreatic Cancer

**DOI:** 10.3390/tomography10100117

**Published:** 2024-10-07

**Authors:** Yusuke Kurita, Daisuke Utsunomiya, Kensuke Kubota, Shingo Koyama, Sho Hasegawa, Kunihiro Hosono, Kuniyasu Irie, Yuichi Suzuki, Shin Maeda, Noritoshi Kobayashi, Yasushi Ichikawa, Itaru Endo, Atsushi Nakajima

**Affiliations:** 1 Department of Gastroenterology and Hepatology, Yokohama City University, Yokohama 236-0004, Japan; kubotak@yokohama-cu.ac.jp (K.K.); t166064d@yokohama-cu.ac.jp (S.H.); hiro1017@yokohama-cu.ac.jp (K.H.); nakajima.ats.dr@yokohama-cu.ac.jp (A.N.); 2 Department of Diagnostic Radiology, Graduate School of Medicine, Yokohama City University, Yokohama 236-0004, Japan; d_utsuno@yokohama-cu.ac.jp (D.U.); singo109@yokohama-cu.ac.jp (S.K.); 3 Department of Gastroenterology, Yokohama City University, Yokohama 236-0004, Japan; k_irie@yokohama-cu.ac.jp (K.I.); suzuki.yui.ar@yokohama-cu.ac.jp (Y.S.); smaeda@yokohama-cu.ac.jp (S.M.); 4 Department of Oncology, Yokohama City University, Yokohama 236-0004, Japan; norikoba@yokohama-cu.ac.jp (N.K.); yasu0514@yokohama-cu.ac.jp (Y.I.); 5 Department of Gastroenterological Surgery, Yokohama City University, Yokohama 236-0004, Japan; endoit@yokohama-cu.ac.jp

**Keywords:** contrast, dual-energy computed tomography, delayed phase, pancreatic cancer

## Abstract

**Background/Objectives**: The usefulness of dual-energy computed tomography (DECT) for low absorption in the parenchymal phase and contrast effects in the delayed phase for pancreatic cancer is not clear. Therefore, the diagnostic capability of low-KeV images obtained using DECT for pancreatic cancer in the pancreatic parenchymal and delayed phases was evaluated quantitatively and qualitatively. **Methods**: Twenty-five patients with pancreatic cancer who underwent contrast-enhanced DECT were included. A total of 50 and 70 KeV CT images, classified as low-keV and conventional CT-equivalent images, were produced, respectively. The tumor-to-pancreas contrast (Hounsfield units [HU]) in the pancreatic parenchymal and delayed phases was calculated by subtracting the CT value of the pancreatic tumor from that of normal parenchyma. **Results**: The median tumor-to-pancreas contrast on 50 KeV CT in the pancreatic parenchymal phase (133 HU) was higher than that on conventional CT (68 HU) (*p* < 0.001). The median tumor-to-pancreas contrast in the delayed phase was −28 HU for 50 KeV CT and −9 HU for conventional CT (*p* = 0.545). For tumors < 20 mm, the tumor-to-pancreas contrast of 50 KeV CT (−39 HU) had a significantly clearer contrast effect than that of conventional CT (−16.5 HU), even in the delayed phase (*p* = 0.034). **Conclusions**: These 50 KeV CT images may clarify the low-absorption areas of pancreatic cancer in the pancreatic parenchymal phase. A good contrast effect was observed in small pancreatic cancers on 50 KeV delayed-phase images, suggesting that DECT is useful for the visualization of early pancreatic cancer with a small tumor diameter.

## 1. Introduction

Pancreatic cancer is a malignant tumor with a poor prognosis, and its incidence has increased in recent years [1,2]. Early detection has an evident advantage in patient management, and imaging modalities with higher tumor detectability are important for improving the clinical outcomes in patients with pancreatic cancer. Although surgical resection is the only curative treatment, detection of early-stage pancreatic cancer amenable to resection remains difficult. The disease is often diagnosed at an advanced stage, and resectable pancreatic cancer accounts for only 10–20% of all cases [3,4]. The effectiveness of multiphasic dynamic contrast-enhanced computed tomography (CT) has been reported, and early-phase imaging is important for the detection of pancreatic hypoattenuating tumors, such as pancreatic cancer. However, detection is often difficult, mainly because of the small size of tumors or the insufficient contrast between the pancreatic tumor and pancreatic parenchyma (tumor-to-pancreas contrast) [5,6]. Although the usefulness of endoscopic ultrasound [7] and magnetic resonance imaging [8] has also been reported, early diagnosis of pancreatic cancer remains difficult.

Low-kilovoltage CT is effective for increasing iodine contrast enhancement because the fraction of photons in the energy of the K-edge of iodine at 33.2 keV is the highest [9], and the CT number of the iodinated contrast material becomes significantly higher in low kV CT than in conventional-kilovoltage CT (120 kVp) [10]. However, the quality of low kV CT images may be impaired by increased noise. In dual-energy CT (DECT), virtual monochromatic images are available at multiple kiloelectron voltage (KeV) levels, starting from 40 KeV, and include both low- and conventional-kilovoltage-equivalent images. It is expected that the lower KeV (<70 KeV) images produced from DECT would theoretically better depict pancreatic tumors with a higher tumor-pancreas contrast compared to conventional CT. The usefulness of low-KeV (<70 KeV) images obtained from DECT for pancreatic tumors has been reported [11,12,13,14,15]. The virtual monochromatic images derived from DECT on the detectability of pancreatic cancer have not been thoroughly investigated.

It is widely known that pancreatic cancer is generally recognized as a hypovascular lesion in pancreatic parenchymal phases on dynamic CT imaging, but recently, hypervascular lesions have been noted to occur in delayed phases with increased sensitivity [16]. However, the characteristics of pancreatic cancer with high absorption changes owing to delayed tumor staining have been unclear. Furthermore, in assessing the feasibility of surgical resection, evaluation of the primary tumor and the involvement of local vessels such as the celiac artery, superior mesenteric artery and vein, portal vein, and hepatic artery are critical for determining resectability [17]. Arterial vascular invasion on low KeV portal-venous-phase CT was evaluated in a previous study [18]; however, there have been no reports investigating the effects of virtual monochromatic images derived from DECT on the vascular invasion in pancreatic parenchymal and portal-venous-phase phase CT images.

Therefore, in this study, the diagnostic capability of low KeV images derived from contrast-enhanced DECT for pancreatic cancer by comparing them with conventional KeV CT images in the pancreatic parenchymal and delayed phases were evaluated quantitatively and qualitatively. The vascular invasion of low KeV images derived from contrast-enhanced DECT was also assessed.

## 2. Materials and Methods

### 2.1. Study Population

**Figure 1:**
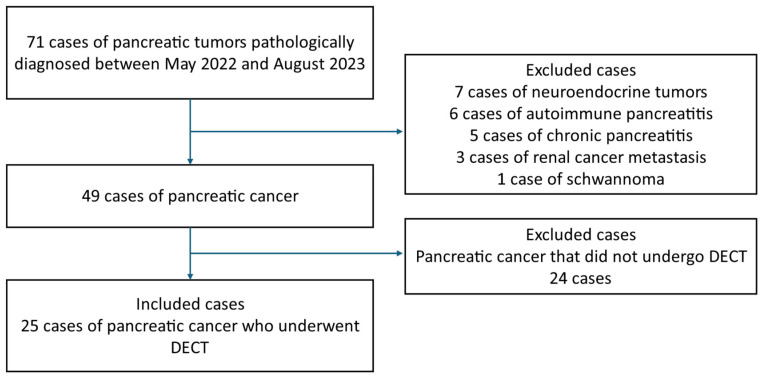
The patient introduction flowchart.

Clinical data were retrospectively collected from Yokohama City University Hospital between May 2022 and August 2023. Patients with pathologically confirmed pancreatic cancer who underwent a contrast-enhanced DECT were included. The pathological diagnosis was based on surgical and ultrasound-guided fine-needle aspiration specimens. The data collected included sex, age, body mass index, tumor size, and tumor location. In this study, ordinary pancreatic cancer was included, and cases that were found to be special types of pancreatic cancer, such as neuroendocrine tumors and autoimmune pancreatitis, were excluded. The patient introduction flowchart is shown in Figure 1.

This study was approved by the Institutional Review Board of Yokohama City University Hospital (B200900001). In this retrospective study, only medical data were used, and the privacy of the participants was upheld.

### 2.2. Dual-Energy Computed Tomography (CT) Data Acquisition

Dual-energy CT examinations were performed using a 320-detector row CT scanner (Aquilion ONE Prism; Canon Medical Systems, Otawara, Japan). Unenhanced CT was also performed. For contrast-enhanced dynamic CT, DECT was performed using a rapid kV-switching technique from high (135 kVp) to low (80 kVp) kilovoltage as the tube detector rotated around the patient. Automatic tube current modulation was applied.

An iodinated contrast medium (600 mg I/kg; Iomeron 350; Bracco, Tokyo, Japan) was injected over a fixed duration of 25 s via a 20-gauge intravenous catheter placed in the antecubital vein. Synchronization between the contrast agent flow and image acquisition was achieved using a computer-assisted bolus-tracking system. CT attenuation values were monitored by a radiology technician. The trigger threshold for the region of interest (ROI) in the descending aorta was set to 200 HU. Four-phase CT acquisition (pancreatic parenchymal, portal, venous, and delayed) was performed. Pancreatic parenchymal and portal phase imaging were initiated 20 and 40 s after the trigger, respectively. Venous- and delayed-phase imaging was performed 120 and 300 s after the initiation of contrast injection, respectively. Virtual monochromatic-energy CT images were reconstructed at 50 and 70 KeV. The 70 KeV CT images were considered equivalent to the conventional 120 kVp CT images. All CT images were reconstructed by using a hybrid iterative reconstruction (Spectral Body Standard, Canon Medical Systems, Tokyo, Japan) with a reconstruction section thickness and interval of 0.5 and 0.5 mm, respectively.

### 2.3. Quantitative Image Analysis

Hounsfield units (HU) were measured by placing an ROI between the tumor and adjacent non-tumor normal pancreatic parenchyma for objective measurements in the pancreatic parenchymal and delayed phases. ROI placement was performed while avoiding vessels, bile ducts, pancreatic ducts, local lesions, and artifacts, and the CT values of the tumor and normal pancreatic parenchyma were measured in each case using the ROI. The tumor-to-pancreas contrast (HU) was calculated by subtracting the CT value of the pancreatic tumor from the CT value of the normal pancreatic parenchyma.

**Figure 2:**
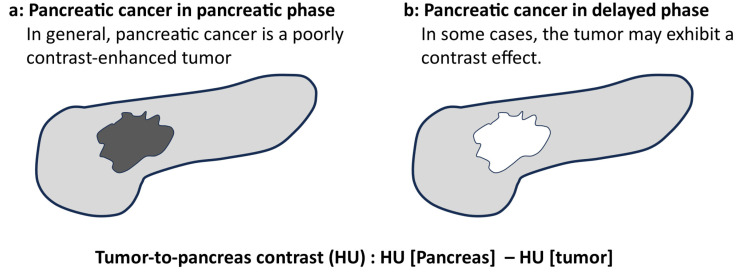
The overview of the quantitative evaluation (created by the author). (**a**) Pancreatic cancer in the pancreatic phase. In general, pancreatic cancer is depicted as a hypoenhanced tumor on CT. (**b**) Pancreatic cancer in the delayed phase. In some cases, the tumor is depicted as a hyperenhanced tumor on CT.

Figure 2 presents an overview of the quantitative evaluation. In general, the pancreatic parenchymal phase has a lower absorption value in the tumor area than in the normal pancreatic parenchyma in pancreatic cancer. Therefore, the tumor-to-pancreas contrast is generally positive in the pancreatic parenchymal phase because it subtracts the CT number of the tumor from that of the normal pancreatic parenchyma. However, pancreatic cancer may show a higher absorption than the normal pancreatic parenchyma in the delayed phase, and the tumor-to-pancreas contrast may be negative. If the tumor has low absorption in the delayed phase, the tumor-to-pancreatic contrast is considered positive.

The contrast difference between the tumor area and normal pancreatic parenchyma (tumor-to-pancreas contrast [HU]) between 50 KeV (low-kilovoltage CT) and 70 KeV CT (conventional-kilovoltage-equivalent image) obtained by DECT was also evaluated. In this study, 70 KeV images were used as conventional CT (120 kVp-equivalent) images for comparison [19]. The tumor-to-pancreas contrast in the pancreatic parenchymal and delayed phases was evaluated. The tumor-to-pancreas contrast based on the tumor diameter was also evaluated.

### 2.4. Qualitative Image Analysis

CT scans for tumor and vascular invasion were evaluated independently by a radiologist (DU: observer 1 with 27 years of experience) and a gastroenterologist (YK: observer 2 with 11 years of experience).

The grading for the diagnosis of pancreatic cancer by CT was defined. The visual evaluation for the presence of the pancreas lesion was graded according to a 5-point scale as follows: 5 (excellent) = the contrast of the tumor and the background pancreatic parenchyma is clear, providing a precise and confident evaluation for the diagnosis of pancreas cancer; 4 (good) = the contrast of the tumor and the background pancreatic parenchyma is commendable, providing useful information for the diagnosis of pancreas cancer; 3 (fair) = the contrast of the tumor and the background pancreatic parenchyma is discernible but partially unclear, providing useful information for the diagnosis of pancreas cancer; 2 (restricted) = the contrast of the tumor and the background pancreatic parenchyma is insufficient for a confident and definitive evaluation; and 1 (poor) = the pancreatic lesion is not visualized, indicating minimal or negligible information for the diagnosis of the pancreas cancer.

The visual evaluation for the vascular invasion of the pancreas cancer by a combination of arterial and portal phase CT images was graded according to a 5-point scale as follows: 5 (excellent) = highly detailed images provide comprehensive insights into the presence or absence of vascular involvement, enabling a precise and confident evaluation; 4 (good) = CT imaging demonstrates commendable proficiency in evaluating vascular invasion in pancreatic cancer; 3 (fair) = the utility of CT images in appraising the vascular invasion in pancreatic cancer is of a moderate nature; 2 (restricted) = CT images provide restricted insights into vascular involvement within pancreatic cancer, providing insufficient information regarding the presence or absence of vascular invasion; and 1 (poor) = the pancreatic lesion is not visualized, indicating minimal or negligible information for the vascular invasion of the pancreas cancer. Arterial invasion of the tumor was evaluated in the pancreatic parenchymal phase, and invasion of the portal venous system was evaluated in the portal phase.

The pancreatic tumor and vascular invasion of the arterial and venous systems in the pancreatic parenchymal, portal, and delayed phases of dynamic CT imaging were independently investigated by two reviewers (a gastroenterologist and a radiologist).

### 2.5. Statistical Analyses

Continuous variables with no correspondence were compared using the Mann–Whitney U test. Corresponding continuous variables were compared using the Wilcoxon signed-rank sum test. Statistical significance was set at *p* < 0.05. The intrareader agreement was assessed with kappa coefficients (<0.20, poor; 0.21 to 0.40, fair; 0.41 to 0.60, moderate; 0.61 to 0.80, good; 0.81 to 1.00, excellent). The correlations in tumor diameter and tumor-to-pancreas contrast between the pancreatic tumor and pancreatic parenchyma were analyzed using Spearman correlation coefficients (r_s_), which were defined as the absolute values of the correlation coefficients (0.0 to 0.2, little correlation; 0.2 to 0.4, somewhat correlated; 0.4 to 0.7, fairly correlated; and 0.7 to 1.0; strongly correlated). A value closer to 1 denotes a stronger positive correlation (when one increases, the other also increases), while a value closer to −1 denotes a stronger negative correlation (when one increases, the other decreases). All the statistical analyses were performed using SPSS version 26 (IBM Corp., Armonk, NY, USA).

## 3. Results

### 3.1. Study Population

Table 1 shows the backgrounds of the 25 patients with pancreatic cancer. The median age was 73 years (range: 45–90), including 13 males and 12 females, with a median body mass index of 20.1. The median tumor diameter was 22 mm (range: 10–70). Tumor localization was as follows: head in 11 patients, body in 10 patients, and tail in 4 patients.

### 3.2. Quantitative Parameters

The quantitative parameters are listed in Table 2. The image noise was significantly higher in the 50 KeV CT images for both the pancreatic parenchymal and delayed phases. The median tumor-to-pancreas contrast (range) in the pancreatic parenchymal phase was 133 (78–279) HU for the 50 KeV CT images and 68 (33–116) HU for the conventional CT images (70 keV), with significantly better contrast in the 50 KeV images (*p* < 0.001 *).

**Figure 3:**
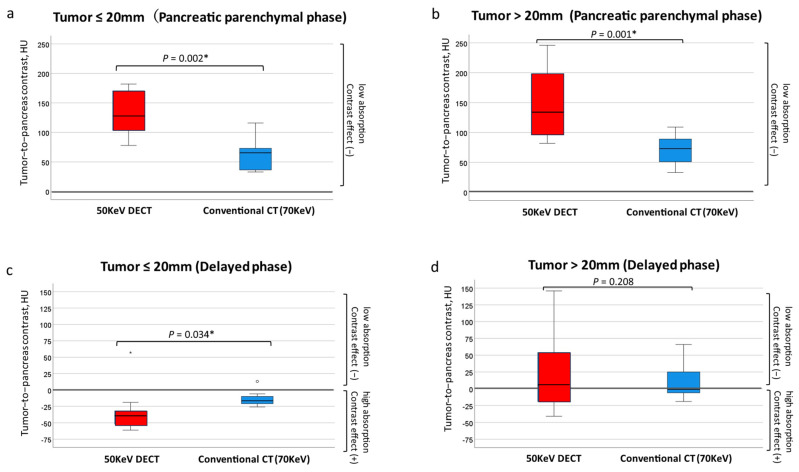
Tumor-to-pancreas contrast (50 KeV DECT vs. conventional CT [70 KeV]). In the pancreatic parenchymal phase CT, the contrast between the tumor and surrounding pancreatic parenchyma was significantly larger at the 50 KeV setting in both ≤20 mm (**a**) and >20 mm lesions (**b**). In the delayed-phase CT, a tumor of ≤20 mm could be detected with significantly higher contrast between the tumor and surrounding pancreatic parenchyma at the 50 KeV rather than 70 KeV setting (**c**), but there was no significant difference in the contrast between 50 KeV and 70 KeV in the >20 mm lesion (**d**). * Statistically significant.

The median tumor-to-pancreas contrast by tumor size in the pancreatic parenchymal phase was significantly larger at 50 KeV DECT for both ≤20 mm and >20 mm (Figure 3). The median tumor-to-pancreas contrast (range) in the delayed phase was −28 (−61–146) HU for the 50 KeV DECT and −9 (−26–66) HU for the conventional CT, with no significant difference (*p* = 0.545). The tumor-to-pancreas contrast in the delayed phase was −39 (−61–57) HU for the 50 KeV CT and −16.5 (−26–13) HU for the conventional CT in tumors 20 mm or smaller, with the 50 KeV CT showing a significantly delayed contrast effect (*p* = 0.034 *) (Figure 3c). There was no significant difference in the tumor-to-pancreas contrast between the two groups for tumors larger than 20 mm (*p* = 0.208) (Figure 3d).

**Figure 4:**
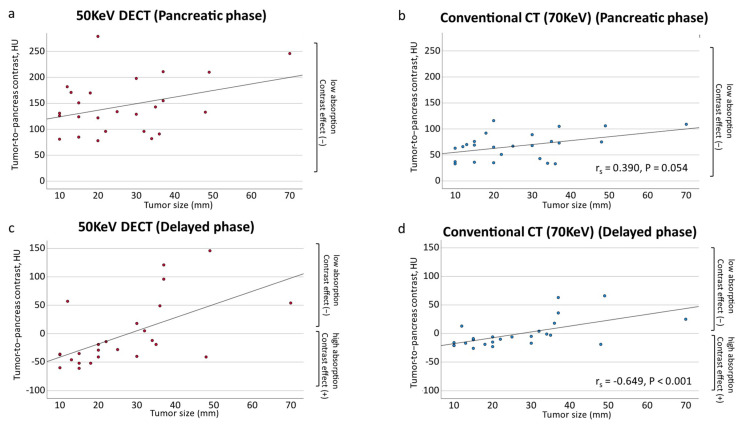
Scatter plots of the contrast difference between tumor and pancreatic parenchyma by tumor diameter. (**a**) Tumor-to-pancreas contrast on 50 KeV DECT in the pancreatic parenchymal phase (r_s_ = 0.270, *p* = 0.192). (**b**) Tumor-to-pancreas contrast of conventional CT (70 KeV) in the pancreatic parenchymal phase (r_s_ = 0.390, *p* = 0.054). (**c**) Tumor-to-pancreas contrast of 50 KeV DECT in the delayed phase (r_s_ = −0.628, *p* < 0.001 *). (**d**) Tumor-to-pancreas contrast on conventional CT (70 KeV) in the delayed phase (r_s_ = −0.649, *p* < 0.001 *). * Statistically significant.

Figure 4 shows a scatter plot of the tumor-to-pancreatic contrast. In the pancreatic parenchymal phase, the tumor-to-pancreas contrast increased with increasing tumor diameter for both the 50 KeV and conventional CT, but the difference was not significant (Figure 4). Figure 4c,d shows the tumor-to-pancreas contrast during the delayed phase. A smaller tumor diameter supports a greater contrast effect observed in the delayed phase. However, as the tumor diameter increased, the contrast effect disappeared, and no contrast effect was observed anymore. Tumors < 20 mm often show delayed-phase contrast effects, whereas tumors > 20 mm often do not. The contrast effect of the delayed phase on the 50 KeV DECT may be useful for the diagnosis of pancreatic cancers smaller than 20 mm. However, for pancreatic cancers > 20 mm in size, the contrast effect of the delayed phase may not be useful for diagnosis.

**Figure 5:**
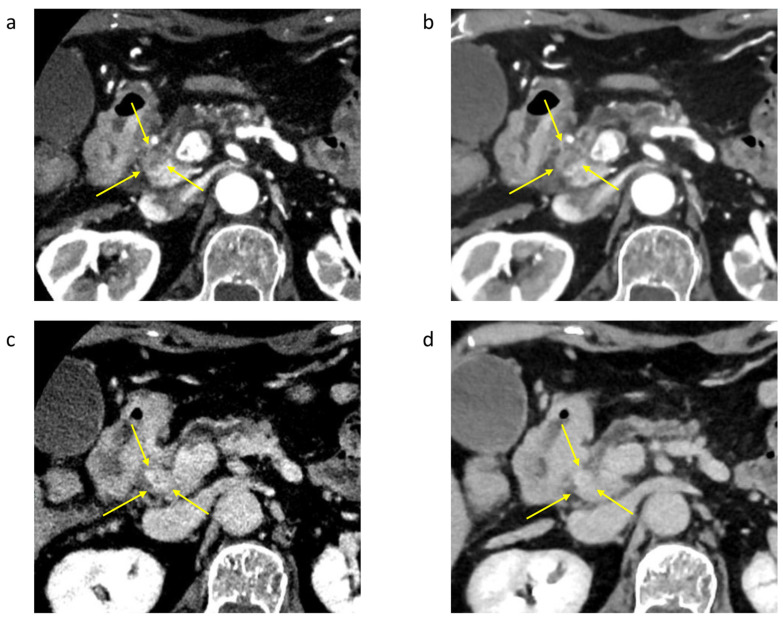
Case of a 77-year-old woman with pancreatic cancer 13 mm in size (Yokohama City University). (**a**) A 50 KeV DECT image was obtained in the pancreatic parenchymal phase. (**b**) A conventional CT image (70 KeV image) was obtained in the pancreatic parenchymal phase. A low-absorption tumor is seen in the area indicated by the arrow. The contrast difference between the tumor and pancreas (tumor-to-pancreas contrast (HU): HU [pancreas] − HU [tumor]) is 171 HU in the 50 KeV DECT image and 70 HU in the conventional CT image (70 KeV), showing the tumor more clearly in the 50 KeV DECT image. (**c**) The 50 KeV DECT image was obtained in the delayed phase. (**d**) A conventional CT image (70 KeV image) was obtained in the delayed phase. The tumor-to-pancreas contrast was −46 HU for the 50 KeV image and −17 HU for the conventional CT image (70 KeV image), indicating that the 50 KeV DECT image clearly showed the contrast effect in the delayed phase.

Figure 5 shows the 50 KeV and conventional CT (70 KeV DECT) images obtained from a representative case. In this case, the tumor was 13 mm in diameter, and the 50 KeV DECT clearly showed a low-absorption area in the parenchymal phase of the pancreas. In the delayed phase, the contrast effect of conventional CT (70 KeV DECT) was unclear; however, the contrast effect of 50 KeV DECT was clear in the tumor area.

### 3.3. Visual Evaluation of Tumor and Vascular Invasion

**Figure 6:**
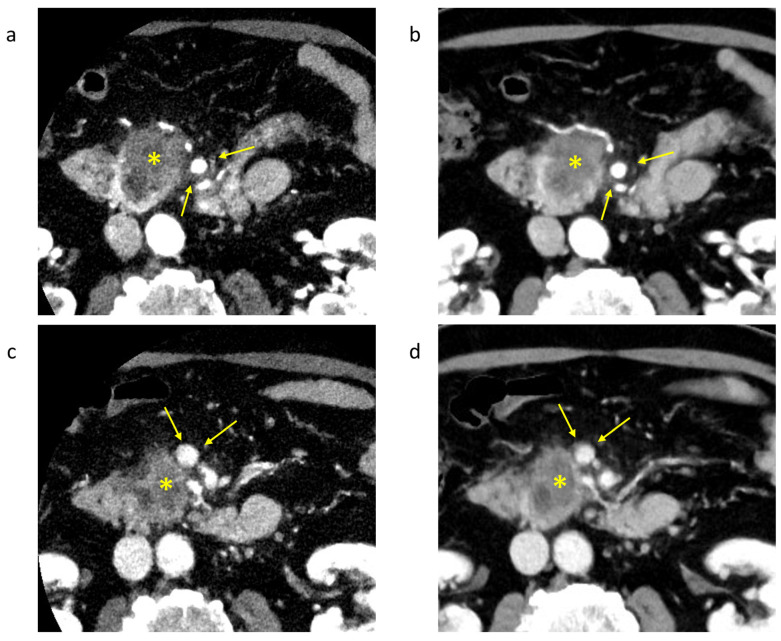
Case of a 75-year-old man with pancreatic cancer (Yokohama City University). *: Tumor area of pancreatic cancer. (**a**) A 50 KeV DECT image was obtained in the pancreatic parenchymal phase. (**b**) A conventional CT image (70 KeV image) was obtained in the pancreatic parenchymal phase. The tip of the arrow is the superior mesenteric artery, which is in contact with the tumor; the 50 KeV DECT image more clearly suggests dorsal involvement of the superior mesenteric artery beyond 180°. (**c**) A 50 KeV DECT image was obtained in the portal phase. (**d**) A conventional image (70 KeV image) was obtained in the portal phase. The tip of the arrow is the superior mesenteric vein, which is in contact with the tumor.

Visualization of the tumor was significantly clearer at 50 KeV than with conventional CT for observers 1 and 2 (Table 3). The contrast of the tumor in the delayed phase also scored higher on the 50 KeV CT images. For vascular invasion, both the arterial and venous portal vessels scored significantly higher on the 50 KeV CT images. A representative case of pancreatic cancer with vascular invasion is shown in Figure 6. The 50 KeV DECT image more clearly suggested dorsal involvement of the superior mesenteric artery beyond 180°. In the 50 KeV DECT image of the portal phase, the tumor was clearly in contact with the superior mesenteric vein. 

The tumor was clearer in the 50 KeV DECT image compared to the 70 KeV image in both the pancreatic parenchymal and portal phases.

Table 4 shows a summary of the similar literature that has previously examined DECT in pancreatic tumors.

## 4. Discussion

It has been reported that low KeV images derived from DECT facilitate tumor and pancreatic evaluation in Table 4 [11,12,13,19,20,21,22]. In this study, the utility of CT images at 50 keV, which is a low keV on DECT, and 70 keV, which is equivalent to conventional imaging, for the diagnosis of pancreatic cancer were compared. Fifty KeV images tended to illustrate pancreatic cancer more clearly. Although the tumor area of pancreatic cancer can generally be identified in the pancreatic parenchymal phase at 70 KeV, which is equivalent to conventional CT, the contrast difference is clearer at 50 KeV, making it easier to distinguish the tumor area. In addition, a 50 keV DECT showed a clearer contrast effect in small pancreatic cancers of 20 mm or less in the delayed phase in this study.

Early pancreatic cancer with a small tumor size is often difficult to diagnose. Compared to CT, endoscopic ultrasound allows for a more detailed observation of smaller lesions [23,24]. EUS also allows tissue sampling [25,26], which is important for tumor differentiation [27]. However, the diagnosis of small pancreatic cancer remains difficult. Recently, small pancreatic cancers have been reported to have increased sensitivity with the addition of delayed-phase CT [16]. In this study, some pancreatic cancers showed good contrast effects in the delayed phase, depending on the tumor type. Compared with the conventional 70 KeV images, 50 KeV images tended to have better contrast in the tumor area in the delayed phase. Although it was previously unknown which pancreatic cancers would demonstrate a contrast effect in the delayed phase, our study results showed that the contrast effect in the delayed phase was unclear in many cases with larger tumors and was more pronounced with tumor diameters of 2 cm or less. The delayed-phase contrast effect may not be useful for diagnosing advanced pancreatic cancer with a large tumor size. In the case of small pancreatic cancers, the contrast effect in the delayed phase may be helpful for the diagnosis of pancreatic cancer. The necrosis and degeneration that may accompany larger pancreatic cancers may be posit, resulting in low-to-isoattenuation of tumors in the delayed phase. Therefore, the DECT of low-KeV images at 50 KeV may reveal a low-absorption area in the pancreatic parenchymal phase and the contrast effect in the delayed phase for the diagnosis of early pancreatic cancer. The dynamic CT of the pancreas with the pancreatic parenchymal and delayed phases obtained at 50 KeV DECT may be recommended if early-stage pancreatic cancer is suspected.

The evaluation of vascular invasion is important for the preoperative assessment of resectability in pancreatic cancer, and the usefulness of vascular invasion of tumors on CT has been reported [28,29]. In addition, evaluation of vascular invasion using MRI [30] and endoscopic ultrasound [29] has been reported. In this study, the ease of evaluating vascular invasion using DECT was investigated. The presence of arterial and venous portal system invasion also tended to be easier to evaluate on the 50 KeV CT than on the conventional 70 KeV CT, suggesting that low KeV DECT images may be easier to evaluate for vascular invasion because of their high contrast. Surgical resection is the only curative treatment for pancreatic cancer and improves prognosis. A more accurate evaluation of vascular invasion using DECT may improve the prognosis of patients with pancreatic cancer.

We adopted 70 KeV CT images as an alternative to standard 120 KVp CT because the 70 KeV CT images show similar CT attenuation of the pancreas [31]. On the other hand, the previous studies showed that the 70 KeV CT images had better image quality than the 120 KVp images due to lower image noise [31,32]. In Canon CT, the image noise can be removed by hybrid-type iterative reconstruction alone, and technical advancement is underway. In our study, 50 KeV images were used, and a comparison between 120 KVp and 70 KeV was not performed. A comparison among 50 KeV (low-kilovoltage monochromatic CT), 70 KeV (conventional-kilovoltage monochromatic CT), and 120 kVp CT (standard-kVp polychromatic CT) should be conducted by using Canon CT. This study was conducted for 50 KeV images, but the optimal energy level is yet to be definitively determined because image noise has been reported to increase in the lowest energy region [19,20,21,22,33]. The Canon CT used in this study has the weakness of having a high noise level. The noise tended to increase when the contrast was set to 50 KeV, but the contrast difference was very large; therefore, the evaluator found these images easy to evaluate. Even if the noise is high, low KeV DECT images may be useful in the diagnosis of pancreatic cancer. It is expected that the combination of DECT and artificial intelligence-based reconstruction algorithms, which are capable of low-KeV imaging with less noise, will become available in the future and may further improve the visualization and accuracy of pancreatic cancer diagnosis. Our study demonstrated that the advantage of DECT was the improved tumor-to-pancreas contrast in the pancreatic and delayed phases, especially in the small lesions. The contrast and detectability of the pancreas tumor in DECT should be compared to those in other imaging modalities, i.e., dynamic MRI and ultrasound.

There have been many recent reports of research using artificial intelligence in the diagnosis of pancreatic tumors, including the evaluation of patients at risk of pancreatic cancer [34], the usefulness of endoscopic ultrasound in the imaging diagnosis of pancreatic cancer [35,36,37], and research on perineural invasion [38]. DECT may be useful for diagnosing small pancreatic cancer, and combining it with artificial intelligence may improve diagnostic performance. There are no reports yet of pancreatic cancer diagnosis using DECT and artificial intelligence, and research on pancreatic tumor diagnosis using DECT and artificial intelligence is expected in the future.

This study has some limitations. First, this was a single-center study with a small number of patients. Second, the low KeV images on DECT used in this study had a lot of noise. Future technical developments in noise reduction in DECT images are desirable. Third, we did not evaluate the image artifacts in DECT. The prior Canon CT used the rotate–rotate (dual-spin) axial KVp-switching mode. Dual-spin and dual-source DECT are more susceptible to temporal and motion misregistration by virtue of the scanner design [39]. In this study, the updated Canon CT machine was used, and the DECT images were obtained by the rapid KVp-switching mode, which consists of quickly and repeatedly switching (<1 ms) from high to low KVp. In the present study population, we noticed no significant misregistration artifacts related to the DECT data acquisition. However, the image quality of the rapid KVp-switching mode might potentially be hampered due to sampling capabilities, and further investigation may be warranted.

## 5. Conclusions

In conclusion, DECT may be useful for visualizing pancreatic cancer. Furthermore, 50 KeV imaging may clarify the low-absorption area in the pancreatic parenchymal phase. In addition, DECT may be more useful for the visualization of early pancreatic cancer with a small tumor size because a clear contrast effect is observed for tumors 20 mm or less on 50 KeV imaging during the delayed phase. DECT at 50 KeV may also facilitate the evaluation of tumor vascular invasion and, consequently, the possibility of surgical resection.

## Figures and Tables

**Table 1:** Patient characteristics.

Characteristic	*n* = 25
Age, median (range), years	73 (45–90)
Sex, male (%)	13 (52.0)
Diabetes (%)	9 (36.0)
Smoking (%)	6 (24.0)
Alcohol intake (%)	12 (48.0)
Body mass index, median (range)	20.1 (16.5–25.8)
Tumor size, median (range), mm	22 (10–70)
Tumor location (%)	
Head	11 (44.0)
Body	10 (40.0)
Tail	4 (16.0)

**Table 2:** Quantitative parameters of 50 KeV and 70 KeV CT images.

	50 KeV CT (Low Kilovoltage)	70 KeV CT (Conventional Kilovoltage)	*p*-Value
Pancreatic parenchymal phase, median (range), HU			
Tumor	114 (36–222)	84 (27–139)	<0.001 *
Normal pancreatic parenchyma	271 (179–398)	152 (103–200)	<0.001 *
Delayed phase, median (range), HU			
Tumor	161 (25–306)	96 (27–154)	<0.001 *
Normal pancreatic parenchyma	155 (100–269)	90 (66–138)	<0.001 *
Noise, median (range)			
Pancreatic parenchymal phase	35 (21–43)	13 (10–17)	<0.001 *
Delayed phase	33 (24–41)	10 (9–15)	<0.001 *
Tumor-to-pancreas contrast, median (range), HU			
Pancreatic parenchymal phase (Total: *n* = 25)	133 (78–279)	68 (33–116)	<0.001 *
≤20 mm (*n* = 12)	238 (78–279)	65 (33–116)	0.002 *
>20 mm (*n* = 13)	134 (82–246)	73 (33–109)	0.001 *
Delayed phase (Total: *n* = 25)	−28 (−61–146)	−9 (−26–66)	0.545
≤20 mm (*n* = 12)	−39 (−61–57)	−16.5 (−26–13)	0.034 *
>20 mm (*n* = 13)	5 (−41–146)	−1 (−19–66)	0.208

CT, computed tomography; HU, Hounsfield units. * Statistically significant.

**Table 3:** Visual evaluation of tumor and vascular invasion in pancreatic cancer.

	50 KeV CT (Low Kilovoltage)		70 KeV CT (Conventional Kilovoltage)		*p*
	Mean ± SD (Range)	κ	Mean ± SD (Range)	κ	
Low contrast effect of pancreatic parenchymal phase					
Observer 1 (Y. K.)	4.72 ± 0.54 (3–5)		3.96 ± 0.84 (2–5)		<0.001 *
Observer 2 (D. U.)	4.72 ± 0.61 (3–5)	0.65	4.20 ± 0.91 (2–5)	0.49	<0.001 *
Contrast effects of delayed phase					
Observer 1 (Y. K.)	3.08 ± 1.08 (1–5)		2.28 ± 0.84 (1–4)		<0.001 *
Observer 2 (D. U.)	3.04 ± 0.84 (2–4)	0.27	2.28 ± 0.54 (2–4)	0.20	<0.001 *
Vascular invasion					
Evaluation of arterial infiltration					
Observer 1 (Y. K.)	4.56 ± 0.77 (2–5)	0.53	4.04 ± 0.84 (2–5)	0.49	<0.001 *
Observer 2 (D. U.)	4.56 ± 0.82 (2–5)		4.16 ± 0.99 (2–5)		0.002 *
Evaluation of venous and portal vascular invasion					
Observer 1 (Y. K.)	4.28 ± 0.61 (3–5)		3.40 ± 1.12 (1–5)		<0.001 *
Observer 2 (D. U.)	4.32 ± 0.99 (2–5)	0.21	3.84 ± 1.07(2–5)	0.19	0.005 *

CT, computed tomography; SD, standard deviation. * Statistically significant.

**Table 4:** Summary of the main similar studies.

Author	Study Design	Patients	CT Scanning Method	Comparison	Sample Size	Conclusions
Bellini, D., 2017, United States [20]	Cohort	Patients with lesions and patients without lesions	Contrast	40, 50, 60, 70, and 80 KeV	59	The maximal contrast-to-noise ratio pancreas occurred at 40 keV
Fujisaki, Y., 2022, Japan [13]	Cohort	Patients with pancreatic cancer and patients without pancreatic tumor	Contrast	40 KeV vs. 120 kVp	112	40 KeV DECT had better sensitivity to small pancreatic cancer
Patel, B., 2013, United States [21]	Cohort	Pancreatic cancer	Contrast	45 KeV vs. 70 KeV	64	Significantly increased pancreatic lesion contrast was noted at 45 KeV
McNamara, M., 2015, United States [19]	Cohort	Patients with small (<3 cm) pancreatic cancer	Contrast	52 KeV vs. 70 KeV	46	The contrast between tumors and non-tumors was greatest at 52 KeV
Aslan, S., 2019, Turkey [22]	Cohort	Pancreatic tumor (cancer, endocrine tumors, other cystic and solid masses)	Contrast	45 KeV vs. 70 KeV	90	The use of low energy levels improves tumor conspicuity
Liang, H., 2023, China [11]	Cohort	Pancreatic tumor (cancer, endocrine tumors, other cystic and solid masse	Non-contrast	Non-contrast and virtual non-contrast images obtained from DECT	106	Virtual non-contrast images of DECT provide diagnostic image quality and accurate pancreatic lesion detection
Noda, Y., 2023, Japan [12]	Cohort	Pancreatic tumor (cancer, endocrine tumors, other cystic and solid masse	Contrast	40 keV vs. 80 kVp	111	40 keV demonstrated higher SNR and tumor-to-pancreas contrast-to-noise ratio compared to the 80 kVp setting.
Kurita, Y., Japan (The present study)	Cohort	Pancreatic cancer	Contrast	50 KeV vs. 70 KeV	25	50 KeV may clarify the contrast between tumors and non-tumors. A delayed contrast effect was observed in small pancreatic cancers on 50 KeV delayed-phase images

## Data Availability

Correspondence and requests for materials should be addressed to Yusuke Kurita.
